# Activity-dependent degeneration of axotomized neuromuscular synapses in *Wld^S^* mice

**DOI:** 10.1016/j.neuroscience.2015.01.018

**Published:** 2015-04-02

**Authors:** R. Brown, A. Hynes-Allen, A.J. Swan, K.N. Dissanayake, T.H. Gillingwater, R.R. Ribchester

**Affiliations:** Euan MacDonald Centre for Motor Neurone Disease Research, Hugh Robson Building, University of Edinburgh, George Square, Edinburgh EH8 9XD, UK

**Keywords:** ALS, amyotrophic lateral sclerosis, ANOVA, analysis of variance, CME, confocal microendoscopy, DL, deep lumbrical muscle, EPP, endplate potential, FDB, flexor digitorum brevis muscle, MEPP, miniature endplate potential, MPS, mammalian physiological saline, NAD, nicotinamide adenine dinucleotide, NMJ, neuromuscular junction, NMN, nicotinamide mononucleotide, Nmnat, nicotinamide mononucleotide adenyl transferase, QC, quantal content, Sarm1, sterile alpha and TIR motif-containing protein-1, SOD, superoxide dismutase, TTX, tetrodotoxin, WD, Wallerian degeneration, Wld^S^, Wallerian degeneration-Slow mutant, WT, wild-type, YFP, yellow fluorescent protein, neuromuscular junction, axotomy, synaptic plasticity, activity

## Abstract

Activity and disuse of synapses are thought to influence progression of several neurodegenerative diseases in which synaptic degeneration is an early sign. Here we tested whether stimulation or disuse renders neuromuscular synapses more or less vulnerable to degeneration, using axotomy as a robust trigger. We took advantage of the slow synaptic degeneration phenotype of axotomized neuromuscular junctions in flexor digitorum brevis (FDB) and deep lumbrical (DL) muscles of Wallerian degeneration-Slow (*Wld^S^*) mutant mice. First, we maintained *ex vivo* FDB and DL nerve-muscle explants at 32 °C for up to 48 h. About 90% of fibers from *Wld^S^* mice remained innervated, compared with about 36% in wild-type muscles at the 24-h checkpoint. Periodic high-frequency nerve stimulation (100 Hz: 1 s/100 s) reduced synaptic protection in *Wld^S^* preparations by about 50%. This effect was abolished in reduced Ca^2+^ solutions. Next, we assayed FDB and DL innervation after 7 days of complete tetrodotoxin (TTX)-block of sciatic nerve conduction *in vivo*, followed by tibial nerve axotomy. Five days later, only about 9% of motor endplates remained innervated in the paralyzed muscles, compared with about 50% in 5 day-axotomized muscles from saline-control-treated *Wld^S^* mice with no conditioning nerve block. Finally, we gave mice access to running wheels for up to 4 weeks prior to axotomy. Surprisingly, exercising *Wld^S^* mice *ad libitum* for 4 weeks increased about twofold the amount of subsequent axotomy-induced synaptic degeneration. Together, the data suggest that vulnerability of mature neuromuscular synapses to axotomy, a potent neurodegenerative trigger, may be enhanced bimodally, either by disuse or by hyperactivity.

## Highlights

• Use and disuse may influence synaptic maintenance but so far evidence for this has been indirect.
• We tested whether stimulation or disuse of neuromuscular junctions in adult *Wld^S^* mice altered vulnerability to axotomy.
• Moderate activity optimized resistance to axotomy while disuse or stimulation increased the rate of synaptic degeneration.

## Graphical abstract

**Figure:**
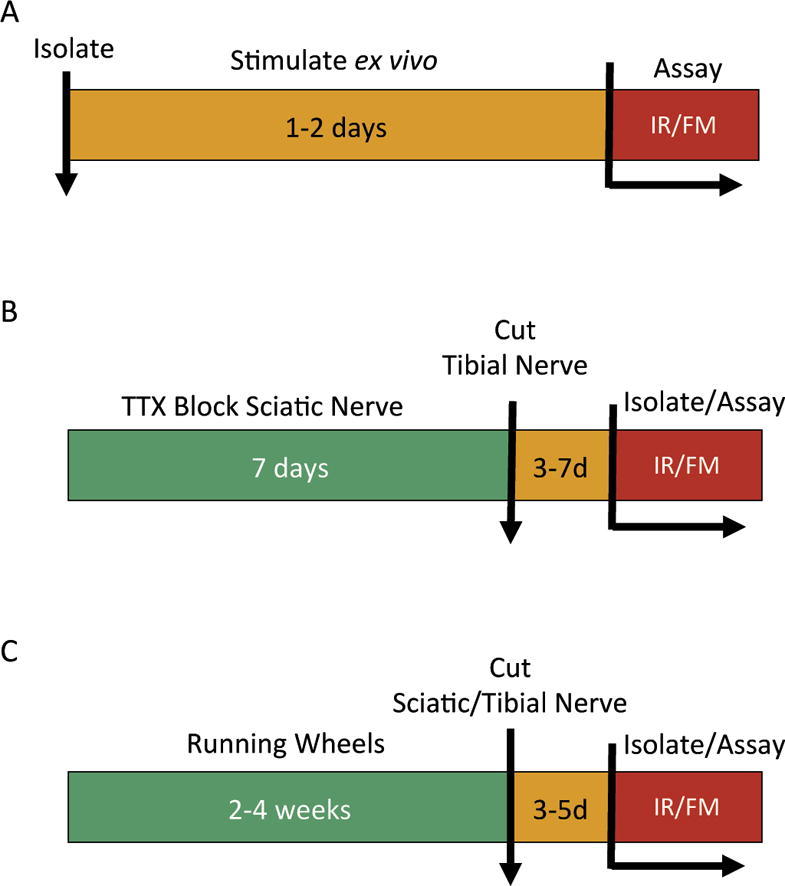

## Introduction

Maintenance and degeneration of synapses are thought to depend on their use or disuse. For instance, activity may influence the rates of synaptic and neuronal degeneration both during normal aging and in neurodegenerative conditions in which cognitive decline or progressive impairment of muscle function is associated with early signs of synaptic dysfunction and demise (Frey et al., 2000; Selkoe, 2002; Swaab et al., 2002; Fischer et al., 2004; Frick and Benoit, 2010; Power et al., 2010; Valdez et al., 2010; Li et al., 2011; Punga and Ruegg, 2012; Stern, 2012; Shors et al., 2012). Altering some forms of neuromuscular activity may also influence progression of disease. For example, imposing moderate levels of activity in mouse models of amyotrophic lateral sclerosis (ALS) delays onset and slow progression of disease signs, reducing premature mortality (Kirkinezos et al., 2003; Veldink et al., 2003; Liebetanz et al., 2004; Deforges et al., 2009; Gordon et al., 2010; de Almeida et al., 2012). By contrast, intensive activity has also been reported to accelerate disease progression, both in mouse models and in sporadic forms of human ALS, perhaps through excitotoxicity or enhancing vulnerability to reactive oxygen species (Carri et al., 2003; Bruijn et al., 2004; Carrasco et al., 2004; Mahoney et al., 2004; Al-Chalabi and Leigh, 2005; Chio et al., 2005; Boillee et al., 2006; Pun et al., 2006; David et al., 2007; Bell and Hardingham, 2011; Alvarez et al., 2013; Mehta et al., 2013).

Despite speculation and debate on the influence of use or disuse of synapses on synaptic degeneration, there is as yet no compelling, direct evidence that connects normal axonal activity to synaptic maintenance, or abnormal axonal activity to the vulnerability of synapses to neurodegenerative triggers. By contrast, there is compelling evidence that some forms of synaptic remodeling or withdrawal are highly sensitive to activity. For instance, the rate of postnatal synapse elimination, a controlled process of presynaptic withdrawal that has been well characterized at developing neuromuscular junctions (NMJs) or following nerve regeneration in adults, is readily modifiable and strongly influenced by activity (Brown et al., 1976; Thompson et al., 1979; Betz et al., 1980a; Ribchester and Taxt, 1983; Taxt, 1983; Thompson, 1983; Barry and Ribchester, 1995; Costanzo et al., 2000; Keller-Peck et al., 2001; Gillingwater and Ribchester, 2003; Walsh and Lichtman, 2003; Personius et al., 2007; Leslie and Nedivi, 2011; Favero et al., 2012; Turney and Lichtman, 2012; Caroni et al., 2014). Reactive growth (sprouting) and enhancement of synaptic function also occurs in adults in response to imposed inactivity or activity (Betz et al., 1980b; Ribchester and Taxt, 1984; Tsujimoto et al., 1990; Dorlochter et al., 1991; Ribchester, 1993; Fahim, 1997). We therefore asked in the present study whether variations in axonal or synaptic activity either before or after a neurodegenerative trigger might influence the resistance of neuromuscular synapses to pathological stimuli for synaptic degeneration in adult muscles as well.

An accessible, reliable and readily controllable model of synaptic degeneration is the Wallerian-like breakdown of motor nerve terminal structure and function that occurs after nerve injury (axotomy) or following functional disruption of axonal transport (Slater, 1966; Miledi and Slater, 1970; Winlow and Usherwood, 1975, 1976; Hudson et al., 1984; Wang et al., 2000; Gillingwater and Ribchester, 2001; Coleman and Freeman, 2010). This process is now thought to have mechanisms in common with several forms of neurodegenerative disease (Conforti et al., 2014). Considerable insight into axotomy-induced degeneration of axons and synapses has been obtained through study of the Wallerian degeneration-Slow mutant (*Wld^S^*) mouse mutant, in which axonal and synaptic degeneration are profoundly retarded by overexpression of a stable, aberrant isoform of the nicotinamide adenine dinucleotide (NAD)-synthetic enzyme nicotinamide mononucleotide adenyl transferase (Nmnat)-1 (Lunn et al., 1989; Ribchester et al., 1995; Mack et al., 2001; Wang et al., 2001; Gillingwater et al., 2002; Gillingwater et al., 2004; Gillingwater et al., 2006; Coleman and Freeman, 2010). This isoform substitutes for a more labile axoplasmic form, Nmnat-2, whose levels decline steeply after axotomy, an event which normally is sufficient to trigger fragmentation and subsequent degeneration of axons (Beirowski et al., 2005; Beirowski et al., 2009; Babetto et al., 2010; Gilley and Coleman, 2010; Gilley et al., 2013; Milde et al., 2013; Di Stefano et al., 2014).

Interestingly, the protective influence of the Wld^S^ protein is modifiable in several ways. For instance, the strength of axonal or neuromuscular synaptic protection in *Wld^S^* mice is sensitive to: housing environment and husbandry of the mice; neuronal maturity, reduced mutant gene copy-number (“gene dose”); disrupted targeting to nuclei or intracellular organelles; and interaction with other genes, including Sarm-1 (Perry et al., 1990; Mack et al., 2001; Gillingwater et al., 2002; Beirowski et al., 2009; Conforti et al., 2009; Wong et al., 2009; Babetto et al., 2010; Osterloh et al., 2012; Massoll et al., 2013). Moreover, motor axons and their terminals are less well protected from axotomy-induced degeneration in *Wld^S^* mice than those of sensory neurons (Brown et al., 1994; Oyebode et al., 2012). There are several plausible explanations for this but a key difference is that sensory endings and their axons continue to be active in response to natural orthodromic stimulation, including touch, pressure, nociceptive and proprioceptive stimuli (Oyebode et al., 2012) whereas distal axons and motor nerve endings, while remaining competent to conduct action potentials and evoke transmitter release, require continuity with their initial segments and motor neuron cell bodies for orthodromic activation, which is interrupted upon axotomy (Tsao et al., 1994; Ribchester et al., 1995; Gillingwater et al., 2002; Beirowski et al., 2009; Brown et al., 2014).

Thus, taking together the indirect evidence for an influential role of activity in normal aging or neurodegenerative disease; the strong influence of activity on the rate of natural remodeling processes like synapse elimination; and the enhanced persistence of sensory endings and their axons observed following axotomy in *Wld^S^* mice, we enquired whether activity also influences Wallerian-like degeneration of axotomized neuromuscular synapses. If this were the case, then we would predict that systematically altering or imposing neuronal activity patterns should change the rate of synaptic degeneration after axotomy.

We tested our hypothesis by controlling axonal and neuromuscular synaptic activity in *Wld^S^* mice, in three ways. First, we measured the effect of continuous stimulation of isolated and cultured nerve-muscle preparations on the rate of synaptic degeneration *ex vivo*. Second, we preconditioned axons by chronic nerve conduction block *in vivo*, then measured the effect of this disuse-priming on synaptic degeneration over several days after axotomy. Finally, we enriched the environment of mice for up to one month, by providing them with running wheels, thus encouraging increased levels of volitional activity, before cutting axons and measuring the subsequent levels of neuromuscular synaptic degeneration. Surprisingly, the data suggest a bimodal response: both inactivity and intense stimulation appear to increase the vulnerability and rate of synaptic degeneration in both *Wld^S^* and wild-type (WT) mice, while moderate levels of activity were beneficial or without adverse effect. We discuss possible implications of the data for unified views of links between Wallerian-like degeneration of neuromuscular synapses, developmental synapse elimination, and neurodegenerative diseases in which synaptic dysfunction or degeneration are early phenomenological or clinical signs (Gillingwater and Ribchester, 2001; Raff et al., 2002; Gillingwater and Ribchester, 2003).

## Experimental procedures

### Ethical approval

All experiments reported in the present paper were approved by the University of Edinburgh College of Medicine and Veterinary Medicine Local Ethics Committee and conducted under the terms of a Project Licence and Personal Licences from the UK Home Office, in accordance with requirements of the United Kingdom Animals (Scientific procedures) Act, 1986. At the end of the experiments, mice were swiftly killed by cervical dislocation, in accordance with the UK Home Office regulations, Schedule 1.

### Mice

Age-matched (5–10 weeks old) male and female C57Bl6, *thy1.2YFP16:C57Bl6*, C57Bl*Wld^S^*, *thy1.2YFP16:Wld^S^* and *thy1.2-YFPH:Wld^S^* mice generated by cross-breeding to homozygosity were used throughout these studies (Lunn et al., 1989; Feng et al., 2000; Beirowski et al., 2004; Beirowski et al., 2005; Bridge et al., 2009; Wong et al., 2009; Oyebode et al., 2012; Teriakidis et al., 2012; Hirst and Ribchester, 2013). All mice used were housed in standard light (12-h light 12-h darkness) and temperature conditions, in cages of 6 or fewer. Mice had access to food and water *ad libitum*. The *thy1.2YFP16* transgenic line expresses yellow fluorescent protein (YFP) in all motor neurons, serving as a useful endogenous and non-toxic reporter of axonal and synaptic structure and integrity (Feng et al., 2000; Bridge et al., 2009; Wong et al., 2009). Expression of YFP also does not interfere significantly with Wallerian degeneration (WD) or the *Wld^S^*-protective phenotype (Beirowski et al., 2004; Beirowski et al., 2005; Bridge et al., 2009; Comley et al., 2011). However, *Wld^S^* mice older than 10 weeks were not used in the present study since the protection of synapses by the chimeric Nmnat1-Ube4b protein responsible for the *Wld^S^* phenotype weakens with age (Perry et al., 1992; Tsao et al., 1994; Tsao et al., 1999; Gillingwater et al., 2002; Beirowski et al., 2009; Adalbert et al., 2012).

### Experimental design

**Fig. 1:**
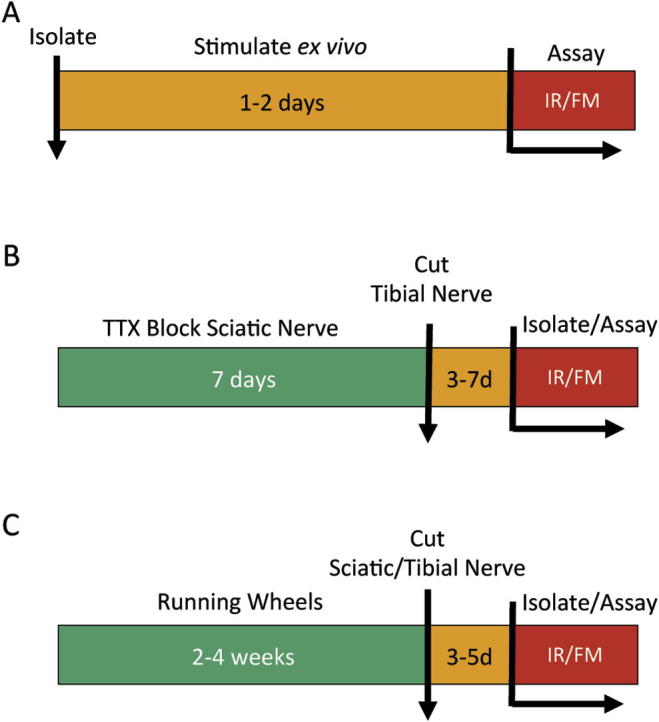
Experimental design. Neuromuscular synapses in *Wld^S^* mouse FDB muscles were challenged by activity and axotomy in three ways. (A) Isolation and *ex vivo* organ culture for 1–2 days, with or without exogenous patterned stimulation to the distal tibial nerve stump, followed by assay using intracellular recording (IR) and/or fluorescence microscopy (FM). (B) Preconditioning sciatic nerve conduction block with tetrodotoxin (TTX) for 1 week, followed by tibial nerve section, then assay of residual innervation. (C) Preconditioned self-motivated activity, by providing mice access to running wheels, for 2–4 weeks, followed by either sciatic or tibial nerve section, then assay 3–5 days later.

The main principle of our experimental design was to use the response of NMJs to axotomy in *Wld^S^* mice as an assay for the effects of either preconditioned or coincident experimental alterations in neuromuscular activity (Fig. 1). Our basic strategy was to use a condition-test approach, in a combination of *in vivo* and *ex vivo* paradigms and *in vitro* physiological and morphological measurements, which has proved successful in exploring modifiers and their mechanisms in other contexts, for instance, axonal degeneration and regeneration (McQuarrie et al., 1977; Boegman et al., 1980; Deshpande et al., 1981; Hudson et al., 1984; Lund et al., 2002; Beirowski et al., 2004; Beirowski et al., 2005; Wong et al., 2009; Oyebode et al., 2012). Our assays were conducted on isolated nerve-muscle preparations of hind toe muscles: specifically flexor digitorum brevis (FDB) or one of the four deep lumbrical muscles (1–4DL). FDB is advantageous for electrophysiological analysis because its muscle fibers are isopotential, obviating the need for precise intracellular positioning of a recording microelectrode (Bekoff and Betz, 1977a,b; Gillingwater et al., 2002; Ribchester et al., 2004). The lumbrical muscles are advantageous for synaptic morphology, because they are thin and the NMJs may thereby readily be prepared and scored rapidly in unsectioned, whole-mounted preparations (Betz et al., 1980b; Gillingwater et al., 2002; Ribchester et al., 2004; Oyebode et al., 2012; Teriakidis et al., 2012; Hirst and Ribchester, 2013). We isolated FDB or 1–4DL nerve-muscle preparations and maintained them for up to 2 days *ex vivo* (that is, in organ culture) at 32 °C, either in the presence or absence of patterned, supramaximal electrical stimulation of their tibial nerve supply (Fig. 1A). In other experiments, we preconditioned the levels of neuromuscular activity in these muscles, supplied by axons in the sciatic nerve, for 1–4 weeks *in vivo*. Nerve conduction was either blocked completely using implants impregnated with tetrodotoxin (TTX; Fig. 1B); or voluntary, self-motivated activity was facilitated by providing mice free access to running wheels fitted in their cages (Fig. 1C). Mice typically elected to run between 1 and 15 km per night with this provision. Neuromuscular synaptic structure and function were evaluated at the end of this period.

### *Ex vivo* assay of synaptic degeneration

We developed a novel *ex vivo* assay for scoring synaptic degeneration during a 48-h period after axotomy (see also (Di Stefano et al., 2014)). We measured the levels of degeneration at different time points in the FDB and lumbrical muscles of four different genotypes of mice: *Wld^S^* (*N* = 27 mice), *thy1.2YFP16:Wld^S^* (*N* = 15), *thy1.2YFP16:Bl6* (*N* = 10) and C57Bl6 (*N* = 12). Mice were sacrificed by cervical dislocation and both hind limbs were removed. The hairy and glabrous skin of both hind feet was stripped. The limbs were then placed in fresh oxygenated mammalian physiological saline (MPS; 120 mM NaCl, 5 mM KCl, 2 mM CaCl_2_, 1 mM MgCl_2_, 0.4 mM NaH_2_PO_4_, 23.8 mM NaHCO_3_, 5.6 mM d-glucose) and equilibrated by bubbling with 95% oxygen/ 5% CO_2_. FDB and lumbrical muscles were dissected, and pinned onto small sheets of Sylgard. They were then placed in sterile 20-ml tubes containing filtered and equilibrated MPS (as above), and maintained at 32 °C in a water bath for 8–48 h. Preliminary experiments established that WT and *Wld^S^* phenotypes could clearly be distinguished after overnight incubation at this temperature. The muscles were bubbled in fresh MPS for 20 min prior to electrophysiological classification of Responsive, Innervated and Unresponsive muscle fibers, as described below. To test for effects of electrical stimulation, FDB muscles were pinned to a Sylgard-lined Petri dish and the nerve was placed in contact with silver wire electrodes integrated into the dish and connected to an external stimulator. Stimulus pulse durations and amplitudes were set to levels that gave visibly strong muscle contractions via neuromuscular transmission. In all cases the stimulus pulses were between 0.1 and 0.5 ms and less than 10V and thus below threshold for direct stimulation of muscle fibers or intramuscular axons. Confirming this, muscles only contracted in response to stimulation when the nerve was placed over the stimulating electrodes. We used three stimulation protocols: 1 Hz continuously, 10 Hz for 1 s every 10 s, and 100 Hz for 1 s every 100 s. Physiological effects were assayed by scoring responsive, innervated and unresponsive muscle fibers after 30 h of stimulation. The effect of varying Ca^2+^ ionic concentration was investigated in MPS containing reduced Ca^2+^ (1 mM) and increased Mg^2+^ (3 mM) in both unstimulated and stimulated muscles. Previous experiments showed that this ratio of Ca^2+^/Mg^2+^ was sufficient to abolish muscle contractions in response to supramaximal stimulation and to reduce mean quantal content (QC) of endplate potential (EPPs) in most FDB muscle fibers to fewer than five quanta (D. Thomson, R. Brown and R.R. Ribchester, unpublished observations.)

#### Electrophysiology

FDB nerve-muscle preparations were dissected and maintained in equilibrated MPS until used for electrophysiological analysis as described above, and previously (Gillingwater et al., 2002; Ribchester et al., 2004). The muscles were pinned in a Sylgard-lined recording chamber. The nerve was stimulated using a glass suction electrode whose inner and outer silver wires were connected to a Digitimer DS2 stimulator (Digitimer, Welwyn Garden City, UK), controlled by a Digitimer D4030 programer. Muscle fiber action potentials were blocked by bathing the isolated preparations in MPS containing 2.5 μM μ-conotoxin (GIIIB μ-CTX; Bachem, Bubendorf, Switzerland) for 20 min, or until muscle twitches in response to nerve stimulation became absent. Glass microelectrodes (1.5 mm o.d. × 0.86 mm i.d., Harvard Apparatus, UK) pulled on a P-87 Flaming/Brown micropipette puller (Sutter Instruments, Novato, CA, USA) were backfilled with 4 M potassium acetate. Microelectrode tip resistances were 30–70 MΩ. Muscle fibers were impaled and EPPs were recorded using an Axoclamp-2B amplifier (Axon Instruments, Molecular Devices, Sunnyvale, CA, USA), low-pass filtered at 3 kHz (Neurolog, Digitimer) and digitized using a CED micro1401 Mk II interface connected to a personal computer (Dell). EPP recordings were analyzed using WinWCP software (Strathclyde electrophysiological software, University of Strathclyde, UK). Spike-2 software (Cambridge Electronic Design, Cambridge, UK) was used to record spontaneous miniature EPPs (MEPPs) and the records were then analyzed using Minianalysis (Synaptosoft, Atlanta, GA, USA). MEPP recordings were only taken from fibers with a resting membrane potential between −60 and −70 mV. Trains of 30 EPPs evoked by supramaximal nerve stimulation at 0.5–2 Hz were recorded from each muscle fiber. From a total of 30 fibers impaled in each muscle, the innervation of fibers was classified by scoring the number of fibers (a) giving an evoked response upon stimulation (“Responsive Fibers”); (b) showing spontaneous MEPPs within a 20–100-s recording period but no evoked response (“Innervated Fibers”); or (c) failing to respond to stimulation and showing no evidence of either evoked EPPs or spontaneous MEPPs in a 20s–100s period of observation (“Unresponsive Fibers”). WinWCP software was used to calculate QC using the Variance Method. The McLachlan–Martin equation: *v*′ = *v*/(1 *−* *fv*/*E*) (p. 322 in (McLachlan and Martin, 1981) in WinWCP was used to correct EPP amplitudes for non-linear summation, assuming the muscle-specific correction factor *f* equal to 0.3; and reversal potential equal to −10 mV.

#### NMJ staining

Lumbrical muscles were normally dissected from the same foot as the FDB preparations, pinned in Sylgard-lined Petri dishes and immersed in MPS containing 5 μg ml^−1^ tetramethylrhodamine-isothiocyanate conjugated with α-bungarotoxin (TRITC-α-BTX; Invitrogen, USA) to label postsynaptic acetylcholine receptors (AChRs). The preparations were then placed on a rocking platform (Stuart Scientific, Chelmsford, UK) for 10 min, washed twice with MPS for 10 min, fixed in 4% paraformaldehyde (PFA, Electron Microscopy Sciences, Hatfield, PA, USA) for 15 min then washed twice with MPS for 10 min. Once fixed and stained, the muscles were mounted on slides using Vectashield (Vector Laboratories, Peterborough, UK) and imaged on an Olympus BX50WI upright fluorescence microscope fitted with a Hamamatsu Orca-ER camera, captured and processed using OpenLab (Improvision/Perkin-Elmer, Coventry, UK) and Adobe Photoshop (USA) software running on an Apple Mac G5 computer. Confocal images were obtained using a BioRad Radiance 2000 confocal microscope via a Nikon Eclipse EFN600 upright microscope, captured and processed using Zeiss Lasersharp software. Motor endplates were scored as “Occupied” when the TRITC-α-bungarotoxin-labeled endplate showed any coverage with a YFP-labeled axon. Endplates with no overlying, YFP-positive axonal input were scored as “Vacant” (that is, denervated).

### Effects of inactivity

These experiments were performed on the following groups of mice: *Wld^S^* (*N* = 11 mice), double-homozygous *thy1.2YFP16:Wld^S^* (*N* = 32), and *thy1.2YFP16:C57Bl6* (*N* = 1) and C57Bl6 (*N* = 4).

#### TTX microcapsule manufacture and implantation

TTX-impregnated microcapsules were manufactured essentially by the method described previously (Martinov and Nja, 2005). Briefly, electrode glass (1.2 mm × 0.94 mm Harvard apparatus, UK) was pulled on a P87 Flaming/Brown micropipette puller. The tip was then broken and polished to a smooth tip using a micro forge (Narishige, Japan) to produce a tip with an external diameter of approximately 200 μm. The other end of the electrode was then scored with a diamond pencil and broken to obtain an overall length of 5 mm, then smoothed using an ethanol lamp. TTX powder in citrate buffer (Alomone Labs, Jerusalem, Israel) was dissolved in sterile saline (Hameln Pharmaceuticals, Gloucester, UK) containing 200 μg/ml Ampicillin (Sigma Aldrich, Dorset, UK) to a final concentration of 15 mM. The microcapsules were filled by capillary action with solutions containing either this solution or the vehicle solution, without TTX, alone. The larger end was then sealed with a small amount of dental wax (Kemdent, Swindon, UK). The tip was protected by inserting it into a small length of polythene tubing (i.d. 2 mm, SF Medical, Hudson, MA, USA).

Prior to surgery, *Wld^S^*, *thy1.2 YFP16: Wld^S^* or C57Bl6 mice aged 5–8 weeks, were prepared with a subcutaneous injection of Vetergesic (0.05 mg kg^−1^). Animals were anesthetized via isoflurane and oxygen inhalation (Merial Animal Health, UK, 2–5%, 0.4–1 l min^−1^). A skin incision was made at the level of the sciatic notch. The underlying muscle was parted by blunt dissection to reveal the sciatic nerve, which was then enclosed within a small cuff made from polythene tubing (i.d. 3 mm, SF Medical, USA). A TTX-filled microcapsule was dipped in sterile saline to remove any excess TTX from the exterior and the tip was then secured inside the cuff. In initial experiments, the tip was inserted into the epineurium but subsequently we found that the block was as effective when the tip of the micropipette was merely lodged inside the cuff. Since this procedure also reduced the risk of nerve damage, we adopted it for most of the experiments.

Nerve conduction block was monitored daily in four ways: (i) by observing the animals weakened grip strength on cage bars, (ii) abnormal walking gait; (iii) absence of a toe-spreading reflex on lifting by the tail; and (iv) absence of a nociceptive reflex response to firm, brief pinching of the foot pad with blunt-tipped forceps. Absence of the toe-spreading reflex and unresponsiveness to firm toe-pinches have been previously shown to be a reliable indicator of effective and complete nerve conduction block (Brown and Ironton, 1977; Lavoie et al., 1977; Betz et al., 1980b; Ribchester, 1993; Barry and Ribchester, 1995; Costanzo et al., 2000). We confirmed this in one anesthetized *Wld^S^* mouse in which the sciatic nerve was stimulated above and below the level of application of the TTX-capillary. Foot muscle twitching was observed upon stimulating distal to the block and there was no response to nerve stimulation above the location of the implant.

#### Tibial nerve axotomy

Animals were anesthetized and the skin of either one or both hindlimbs was swabbed with sterilizing solution as described above. The tibial nerve was exposed by blunt dissection and a small section was removed to delay regeneration. Wounds were sutured using 7-0 Mersilk suture (Ethicon Products, Livingston, UK). Mice were killed 3–7 days later by cervical dislocation and electrophysiological and morphological studies were performed using the methods described above. In C57Bl6 mice the level of degeneration was assessed 12 h after nerve cut. In some cases the contralateral side was used as an unoperated control.

#### Live imaging of axonal and synaptic degeneration

A handheld fiber-optic confocal microendoscope (Cellvizio, Mauna Kea Technologies, Paris) was used to monitor axonal and synaptic degeneration *in vivo* in YFP expressing transgenic mice (Wong et al., 2009; Brown et al., 2014). A small incision was made in the hindlimb of the anesthetized mouse, permitting access to a Proflex S-1500 probe, with a tip diameter of 1500 μm. The inbuilt diode laser provided fluorescence excitation at 488 nm. Emitted light of 505–700 nm was collected through the same optical fibers. Tibial nerves and surrounding muscles were exposed and visualized at up to 7 days post-axotomy in mice with and without preconditioning TTX-block of the sciatic nerve. Stills were obtained from the videos via ImageCell software, and nerve fragment length measured using ImageJ (downloadable from *http://rsb.info.nih.gov/ij*)*.*

### Effects of running wheel activity

To facilitate activity *in vivo*, heterozygous or homozygous *Wld^S^* mice were individually housed in polycarbonate cages fitted with running wheels, either for 2 weeks (*N* = 10) or 4 weeks (*N* = 7). The wheels were connected to revolution counters and average daily running distance was calculated from the number of complete wheel revolutions. In additional *Wld^S^* mice (*N* = 8) and C57Bl6 mice (*N* = 8) mice, circadian wheel-running activity was monitored using Clocklab software (Actimetrics, Wilmette, IL, USA) as described previously (Shen et al., 2000; Harmar et al., 2002). Control *Wld^S^* mice (*N* = 6) were individually housed with no additional environmental enrichment. Following the defined self-motivated running period, mice were anesthetized with isoflurane and the sciatic nerve sectioned on one side, as described above. A small (ca. 2 mm) piece of sciatic nerve was removed to inhibit regeneration. Five days later electrophysiological analysis in isolated FDB nerve-muscle preparations was used to score responsive and innervated fibers as described above. The animals were matched for age at the time of sacrifice.

### Statistical analysis

Statistical analysis was performed using GraphPad Prism software (GraphPad Software Inc, San Diego, CA, USA), using unpaired *t*-tests for comparing two groups of continuous data and an analysis of variance (ANOVA, with post hoc tests as indicated) for multiple groups. All data are represented as mean ± SEM, unless otherwise stated. ‘*N*’ refers to the number of animals, whereas ‘*n*’ represents the number of muscles.

## Results

We tested for influences of activity on synaptic degeneration at *Wld^S^* mouse NMJ in three ways. First, we isolated nerve-muscle preparations and stimulated them at different frequencies for up to 48 h *ex vivo* (Fig. 1A). Second, we tested the influence of inactivity on synaptic degeneration, by conditioning sciatic nerve axons with chronic conduction block for 7 days *in vivo*, then we measured the rate of synaptic degeneration 3–7 days after cutting the inactivated axons (Fig. 1B). Finally, we tested whether enriching the behavioral environment of the mice for 2–4 weeks would influence the protection of their neuromuscular synapses from axotomy-induced degeneration, by providing cage access to running wheels before triggering synaptic degeneration by axotomy (Fig. 1C).

**Fig. 2:**
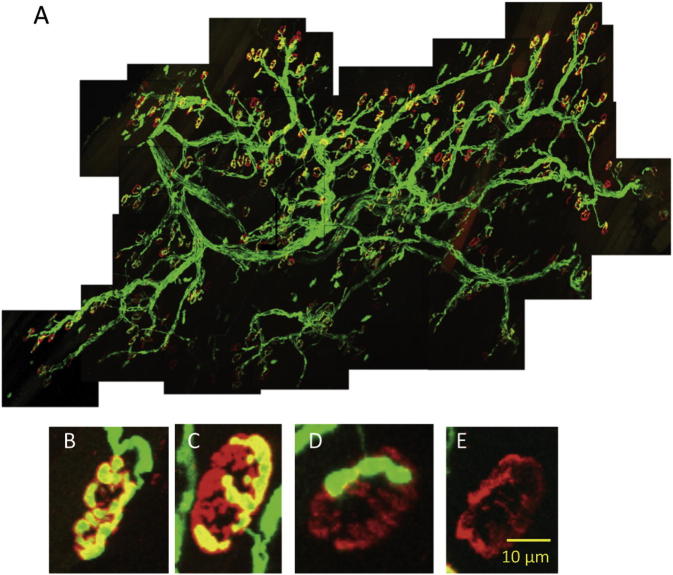
Synaptic degeneration in axotomized *Wld^S^* mouse muscles is asynchronous. (A) Whole-mount montage of a *thy1.2YFP16:Wld^S^* mouse DL muscle, 5 days after cutting the tibial nerve. YFP fluorescence (green) and TRITC-α-BTX (red) counterstaining of AChR at motor endplates shows many NMJ were still fully occupied (B). Partially occupied (C, D) and vacant (denervated) endplates (E) were also readily discernible. Scale bar in E applies to images B–E. (For interpretation of the references to color in this figure legend, the reader is referred to the web version of this article.)

**Fig. 3:**
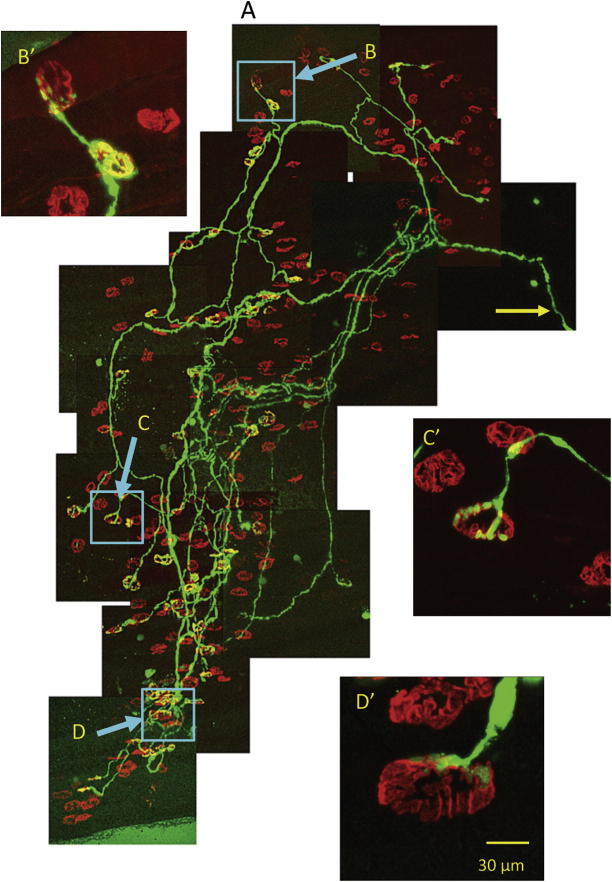
Synaptic degeneration within axotomized motor units of *Wld^S^* mouse muscle is also asynchronous. (A) Whole-mount montage of a single fluorescent motor unit in a *thy1.2YFPH:Wld^S^* mouse DL muscle, 5 days after tibial nerve section. Only about 5% of motor units express YFP in the YFP-H transgenic line. In this, fortuitous instance, only a single unit (axon indicated by yellow arrow) was fluorescent. In this unit, some NMJs were still fully occupied (B, B′); others were partially occupied (C, C′) or almost vacant (D, D′). (For interpretation of the references to color in this figure legend, the reader is referred to the web version of this article.)

As our initial benchmark, we first established the pattern of innervation of toe muscles in *Wld^S^* mice 5 days after axotomy *in vivo*, utilizing co-expression of the thy1.2-YFP transgene as a reporter. Whole-mounts of DL muscles showed a pattern of fully occupied, partially occupied and vacant motor endplates, suggesting that degeneration occurs asynchronously within the population of NMJs (Fig. 2). Quantitative scoring of endplate occupancy and functional responses in *thy1.2YFP16:Wld^S^* mice showed that about 25–50% of endplates were denervated by 5 days post axotomy, which is a similar incidence at that time point to immunostained material (Gillingwater et al., 2002; Bridge et al., 2009). Inspection of single axotomized motor units in *thy1.2YFPH:Wld^S^* mice, in which only about 5% of motor neurons express YFP, revealed occupied and partially vacated endplates within the same motor unit (Fig. 3), suggesting that synaptic retraction occurs asynchronously even within motor units and may therefore be locally regulated (Keller-Peck et al., 2001; Gillingwater and Ribchester, 2003).

### Synaptic degeneration *ex vivo* is accelerated by high-frequency stimulation

We tested directly the effects of activity on synaptic degeneration, by isolating tibial nerve/FDB and DL muscle preparations and stimulating them via their nerve supply, for up to 48 h, while they were bathed in MPS (see Fig. 1A).

**Fig. 4:**
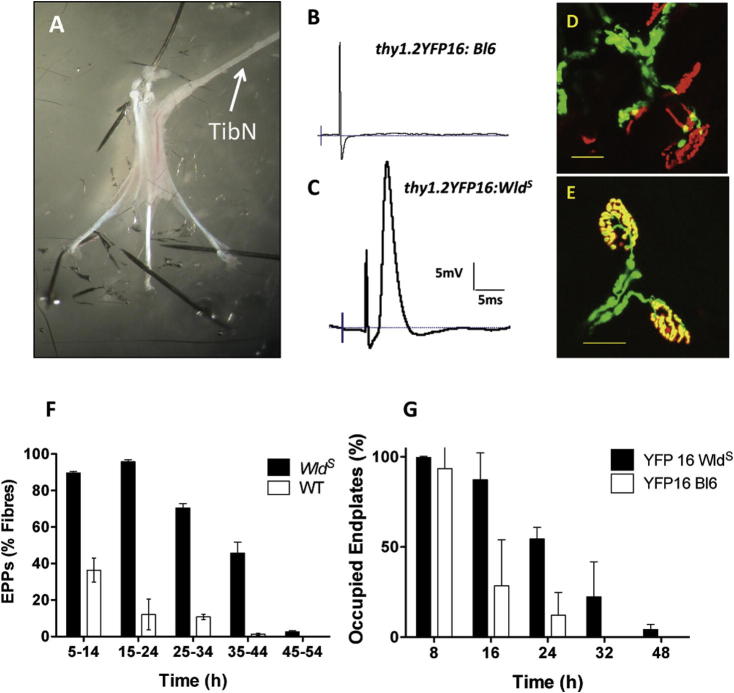
Culturing FDB muscles *ex vivo* resolves *Wld^S^* and control phenotypes. (A) Tibial nerve (TibN)/FDB muscle preparation pinned to a Sylgard chamber, used for the *ex-vivo* culture and subsequent electrophysiological analysis. (B, C) Representative electrophysiological recordings from wild-type (B) and *Wld^S^* (C) FDB muscle fibers, after 24 h culture *ex vivo*. (D, E) Confocal images of motor nerve terminals from *thy1.2YFP16C57:Bl6* (D) and *thy1.2YFP16:Wld^S^* (E) lumbrical muscles, 24 h after *ex vivo* culture at 32 °C. Images digitally adjusted for overall brightness and contrast only. (F) Time course of loss of innervation as measured electrophysiologically from the incidence of FDB muscle fibers responding with EPPs to nerve stimulation in *Wld^S^* (filled bars) and wild-type mouse muscles. (G) Comparable incidences of occupied NMJ assayed in DL muscles morphologically using fluorescence microscopy. Both graphs show mean ± S.E.M., in *n* = 2–17 muscles.

In preliminary experiments we found that persistence of synaptic function  over 12–48h *ex vivo* was strongly temperature dependent. Surprisingly, and by contrast with the rates of synaptic degeneration observed *in vivo*, incubating preparations in MPS at 37 °C rendered synaptic degeneration as rapid in preparations from *Wld^S^* mice as in WT preparations. Conversely, culturing the explanted muscles at 25 °C slowed degeneration of synaptic terminals in WT mice to such an extent that the effect of *Wld^S^* could not be distinguished at this temperature either (data not shown, but see also (Tsao et al., 1999)). However, when muscles from homozygous *Wld^S^* mice were incubated at 32 °C, many NMJ’s were still functional 24 h later, compared with WT muscles in which most did not respond to nerve stimulation (Fig. 4B, C, F). Specifically, in preparations from *Wld^S^* mice, 89.61 ± 2.93% of fibers (*n* = 11 muscles) responded with nerve-evoked EPPs at 5–15 h. After 15–25 h, there was no significant change (95.88 ± 1.15% responsive fibers; *n* = 17) and most NMJs (70.42 ± 7.06%, *n* = 8) still showed significant persistence of synaptic function after 25–30 h *ex vivo* (Fig. 4F). The number responding at 35–45 h was reduced to 45.72 ± 11.90% (*n* = 4). By 45–50 h, substantial numbers of fibers had become denervated but 2.50 ± 1.71% (*n* = 5) still remained functional. By contrast, in WT muscles neuromuscular synapses degenerated much more rapidly: only 36.39 ± 16.02% of NMJs responded to stimulation at 15 h (*n* = 6 muscles), and only 10.77 ± 5.30% at 15–25 h (*n* = 13 muscles). By 25 h, only 1.25 ± 1.25% of fibers produced evoked synaptic responses (*n* = 4 muscles). Thus, 32-h incubation of these nerve muscle preparations *ex vivo* constituted a suitable checkpoint for determining the rate of synaptic degeneration following stimulation in *Wld^S^* muscles, as approximately 50% of synapses were innervated, meaning that potential positive or detrimental effects of stimulation could be distinguished.

Morphological data obtained from *thy1.2YFP16:Wld^S^* and *thy1.2YFP1C57Bl6* mouse DL nerve-muscle preparations corroborated the physiological data (Fig. 4D, E, G). Most NMJ remained occupied by motor nerve terminals up to 24 h after explant of DL nerve-muscle preparations from the *thy1.2YFP16:Wld^S^* line, whereas most in the control *thy1.2YFP1C57Bl6* line had degenerated (Fig. 4G). Specifically, 99.68 ± 0.83% (*n* = 4 muscles) of NMJs in DL muscles from *thy1.2YFP16 Wld^S^* mice were innervated for at least 8 h in MPS. By 16 h, innervation of motor endplates declined slightly, to 87.31 ± 4.13% of NMJs (*n* = 4), then to 54.45 ± 6.50% (*n* = 5) by 24 h. By 32 h, 22.33 ± 19.38% of motor endplates still remained occupied (*n* = 3). By 48 h, only 4.25% of NMJs remained (*n* = 2 muscles). By comparison, NMJs degenerated at least twice as rapidly in explants from the control mice (Fig. 4G). Although almost all intramuscular axons and NMJs in preparations from *thy1.2YFP16:Bl6* mice remained intact for about 8 h (92.46 ± 11.62%, *n* = 7), by 16 h the number of occupied endplates had declined steeply, to 28.51 ± 25.56% (*n* = 6). By 24 h, innervation had further decreased to only 12.09 ± 12.67% (*n* = 7) of motor endplates: less than one-fourth the number in *Wld^S^* preparations. Thus, while the rate of synaptic degeneration *ex vivo* in preparations excised from *Wld^S^* mice appears to be more rapid compared with the rate *in vivo* (Ribchester et al., 1995; Tsao et al., 1999; Gillingwater et al., 2002; Parson et al., 2004), incubation in MPS for 24 h at 32 °C was sufficient to distinguish very clearly the *Wld^S^* from the WT phenotype.

**Fig. 5:**
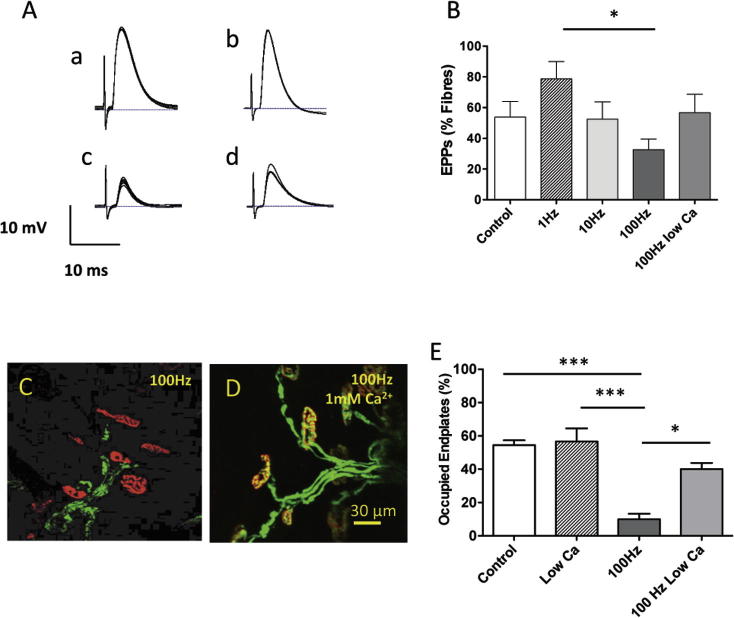
High-frequency patterned stimulation accelerates synaptic degeneration. (A) Representative recordings of EPPs from FDB muscle fibers in (a) unstimulated control; (b) 1 Hz continuously stimulated; (c) 100 Hz stimulated (1s/100s for 32 h); (d) 100 Hz stimulated, same pattern, but with Ca^2+^ reduced to 1 mM and Mg^2+^ increased to 3 mM during the period of stimulation. The assays were all performed in normal MPS. (B) Summary data for responsive fibers, assayed 32 h after axotomy in FDB muscles. The difference between 1 Hz and patterned 100-Hz stimulation is significant (*P* < 0.05; ANOVA/Bonferroni). The differences between the other groups, including the low Ca^2+^ medium, were not significant. (C) NMJ in DL muscles from *thy1.2YFP16:Wld^S^* mice cultured at 32 °C for 24 h, with the tibial nerve supply stimulated at 100 Hz (1 s/100 s) in normal MPS and (D) in MPS with reduced Ca^2+^ and increased Mg^2+^. Images digitally enhanced for overall brightness and contrast only. (E) Summary of morphological analysis of occupied NMJ in control muscles without stimulation in normal MPS, compared with MPS containing reduced Ca^2+^ and increased Mg^2+^, compared with patterned 100 Hz stimulation, in normal versus low Ca^2+^ MPS. The reduced incidence of occupied endplates in the group receiving 100-Hz stimulation was highly significantly different from the other groups, and this effect was abolished in the reduced Ca^2+^/increased Mg^2+^ group (*P* < 0.01 ANOVA/Bonferroni. Graphs in B and E show mean ± S.E.M., *n* = 3–9 muscles. Single asterisks, *P* < 0.05; triple asterisks: *P* < 0.001).

To test whether activity might alter the rate of synaptic degeneration in these *ex-vivo* explants, we stimulated the NMJs at frequencies between 1 and 100 Hz before assaying residual functional innervation (Figs. 1A, 5). We used stimulus patterns that applied an equal number of stimuli to the tibial nerve over this period (either 1 Hz continuously; or 10 Hz for 1 s every 10 s; or 100 Hz for 1 s every 100 s). Examples of EPPs recorded after these stimulation protocols are shown in Fig. 5A. Stimulation at a frequency of 1 Hz appeared to delay synaptic degeneration but the difference was not quite statistically significant (78.75 ± 11.21% vs. 53.75 ± 10.28%, *n* = 4, *t* = 1.76, *P* > 0.05: ANOVA; Fig 5B). Stimulation at 10 Hz for 1 s every 10 s produced no significant difference in the number of responsive fibers compared with unstimulated controls (52.50 ± 11.17%, vs. 53.75 ± 10.28%, *n* = 4, *P* > 0.05: ANOVA). However, stimulation at 100 Hz for 1 s every 100 s accelerated synaptic degeneration. After 32 h, the mean number of responsive fibers was reduced by about one-half, to 32.67 ± 6.86% (*n* = 5 muscles; *P* = 0.056, ANOVA; and Dunnett’s post hoc test: *P* < 0.05 compared with 1 Hz stimulation; *F* = 2.93, df = 19; Fig. 5B). When fibers with MEPPs only were included in the tally, the difference comparing unstimulated controls with patterned 100-Hz stimulation was highly significant (93.33 ± 3.12%, vs. 68.67 ± 6.96%; *t* = 2.953; df = 7; *P* = 0.011; one-tailed *t*-test). We observed a similar effect of stimulation when muscles were assessed morphologically (Fig. 4C, E). In DL muscles from *thy1.2YFP16:Wld^S^* mice maintained in normal MPS and scored after 24 h in culture, 54.45 ± 6.50% of NMJ (*n* = 5 muscles) were occupied by YFP-positive terminals. Muscles stimulated at 100 Hz in normal MPS showed a significant, fivefold reduction in occupancy (9.95 ± 3.33% of endplates, *n* = 4) compared to the unstimulated group (*P* < 0.001; ANOVA/Bonferroni).

The activity-dependent sensitization of synapses to degeneration was inhibited by reducing [Ca^2+^] in the bathing medium. We reduced extracellular [Ca^2+^] for 32 h to 1 mM and increased [Mg^2+^] to 3 mM, then measured physiological responses in FDB muscles after returning them to normal MPS. In unstimulated muscles, the number of responsive fibers was not altered by this change in divalent cation ratio: 53.75 ± 10.28% (*n* = 4 muscles) of fibers responded with EPPs, compared with 52.11 ± 9.11% (*n* = 9) in normal MPS (*P* > 0.05, ANOVA, Fig. 5B). Stimulation at 100 Hz for 32 h in the low [Ca^2+^] medium reduced the subsequent mean incidence of unresponsive fibers compared with that in stimulated muscles incubated in normal MPS but the difference was not quite statistically significant (32.67 ± 6.86% responsive, *n* = 5 vs. 56.67 ± 12.02%, *n* = 3, *t* = 1.67; *P* > 0.05, *t*-test, Fig. 5B)*.* However, the morphological data from cultured DL muscles were more compelling (Fig. 5C–E). As in the FDB muscles assayed physiologically, incubation in low-Ca^2+^ MPS alone, without stimulation, had no effect on degeneration: 56.60 ± 17.85% (*n* = 5) of the motor endplates were occupied after 24 h *ex vivo*, which was not significantly different compared to controls in normal MPS. Incubating muscles in the reduced Ca^2+^/elevated Mg^2+^ medium significantly protected NMJs from the effects of stimulation, leaving 40 ± 6.63% of endplates occupied (*n* = 3; *P* < 0.001; ANOVA/Bonferroni) about four times the number in muscle stimulated at 100 Hz in normal MPS, and not significantly different from the unstimulated controls (Fig. 5E).

These data suggest prolonged and intensive synaptic activity accelerates synaptic degeneration and the mechanism is sensitive to [Ca^2+^]. Thus, the enhanced protection of sensory axons and terminals compared with motor axons and their terminals cannot be simply explained by differences in their ongoing activity post axotomy (Brown et al., 1994; Ribchester et al., 1995; Gillingwater et al., 2002; Oyebode et al., 2012).

In the following two groups of experiments we turned to a different but related issue: that is, whether conditioning axonal and neuromuscular activity influences the vulnerability of synapses to subsequent axotomy-induced degeneration. To address this, we reverted to intact animals and either blocked or enhanced activity levels experimentally *in vivo*.

### Conditioning TTX block accelerated axotomy-induced synaptic degeneration *in vivo*

**Fig. 6:**
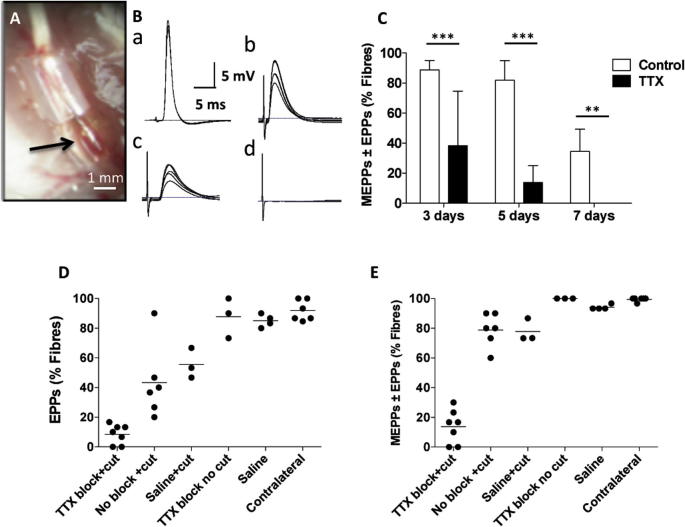
TTX block sensitizes synapses to axotomy. (A) TTX-impregnated capillary (arrow) *in situ*, with its opening secured to the exposed sciatic nerve of a *Wld^S^* mouse, via silicone rubber cuff. (B) Representative intracellular recordings obtained 5 days after axotomy from *Wld^S^* mice: (a) no axotomy (contralateral control); (b) saline implant; (c) TTX block only; (d) TTX-block then axotomy. (C) Time course of loss of functional innervation in axotomized, saline vehicle control muscles (open bars), compared with preconditioning TTX block (filled bars); mean ± S.E.M., *n* = 4–7 muscles per group. (D) Complete data from six groups of mice showing the incidence of responsive fibers, 5 days after axotomy in those indicated as cut. From left to right, the categories were as follows; TTX block + cut: sciatic nerve block for 7 days followed by section of the tibial nerve; No block + cut: tibial nerve section only; Saline + cut: saline vehicle infusion of the sciatic nerve for 7 days, followed by section of the tibial nerve; TTX block no cut: sciatic nerve block for 7 days only; Saline – saline vehicle infusion of the sciatic nerve only; Contralateral – data from muscles on unoperated contralateral sides of the TTX block + cut group. (E) As in C, but complete data including fibers that showed only MEPPs on intracellular recording. TTX block + cut groups are highly significantly different from the other groups (*P* < 0.01, ANOVA/Bonferroni).

In this second group of experiments we conditioned axons and NMJ in *Wld^S^* mice with 7 days of inactivity, achieved by continuous local instillation of TTX from a microcapsule secured to the sciatic nerve (Martinov and Nja, 2005) (see Fig. 6A). We then cut the tibial nerve and assayed synaptic function 3–7 days later. Sample records of evoked EPP responses are shown in Fig. 6B and the time course of degeneration is shown in Fig. 6C. Axotomy triggered synaptic degeneration following 7 days of conditioned inactivity at least twice as rapidly as in muscles that were axotomized but not TTX-blocked beforehand. Three days post axotomy, only 38.34 ± 18.13% of prior TTX-blocked NMJs showed evidence of persistent synaptic transmission, compared with 88.75 ± 3.15% in saline vehicle controls, (*n* = 4 per group). By 7 days, no NMJs in the TTX-blocked group showed evidence of either spontaneous or evoked neurotransmitter release, compared with 34.58 ± 14.74% in the controls (*n* = 4 per group; Fig. 6C; compare for example with (Gillingwater et al., 2002).

In order to rule out influences on degeneration unrelated to activity in these experiments, we compared the percentage of muscle fibers with spontaneous or evoked responses to nerve stimulation with that in the following control groups: vehicle (saline-only) with or without nerve section; TTX-block with no nerve section; and nerve section-only (Fig. 6D, E). About five times fewer fibers produced evoked responses in TTX-conditioning blocked preparations 5 days post axotomy, compared with saline-treated axotomized controls, or axotomized muscles with no nerve implants. In the TTX-blocked and axotomized group: 8.57 ± 2.51% (*n* = 7) of NMJs produced evoked EPPs compared to 55.56 ± 5.88% (*n* = 3) in saline controls and 43.34 ± 10.11% (*n* = 6) in axotomized muscles with no implant (*P* < 0.05, ANOVA, Fig. 6D). When fibers showing only MEPPs were combined with those responding with EPPs, the contrast between TTX pre-conditioned and the other groups was even greater (Fig. 6E).

To test whether microcapsules containing TTX themselves triggered any axonal or synaptic degeneration alone during the conditioning period we implanted microcapsules containing either saline or 15 mM TTX, then FDB muscles were isolated and assayed electrophysiologically 7 days later, without intervening tibial nerve section. There was no evidence of significant synaptic degeneration in these muscles (Fig. 6D, E). The percentage of responsive fibers compared to those in contralateral controls, or those treated with saline were, respectively: TTX-blocked: 87.78 ± 7.78% (*n* = 3 muscles); contralateral controls: 91.88 ± 2.83% (*n* = 6); and saline controls: 85.00 ± 2.15% (*n* = 4; *P* > 0.05, ANOVA).

Morphological analysis of synaptic occupancy corroborated the electrophysiological data. For these experiments we studied axotomized DL muscles in *thy1.2YFP16:Wld^S^* mice, in which all motor neurons and their axons and terminals were fluorescent (Feng et al., 2000; Wong et al., 2009; Teriakidis et al., 2012).

**Fig. 7:**
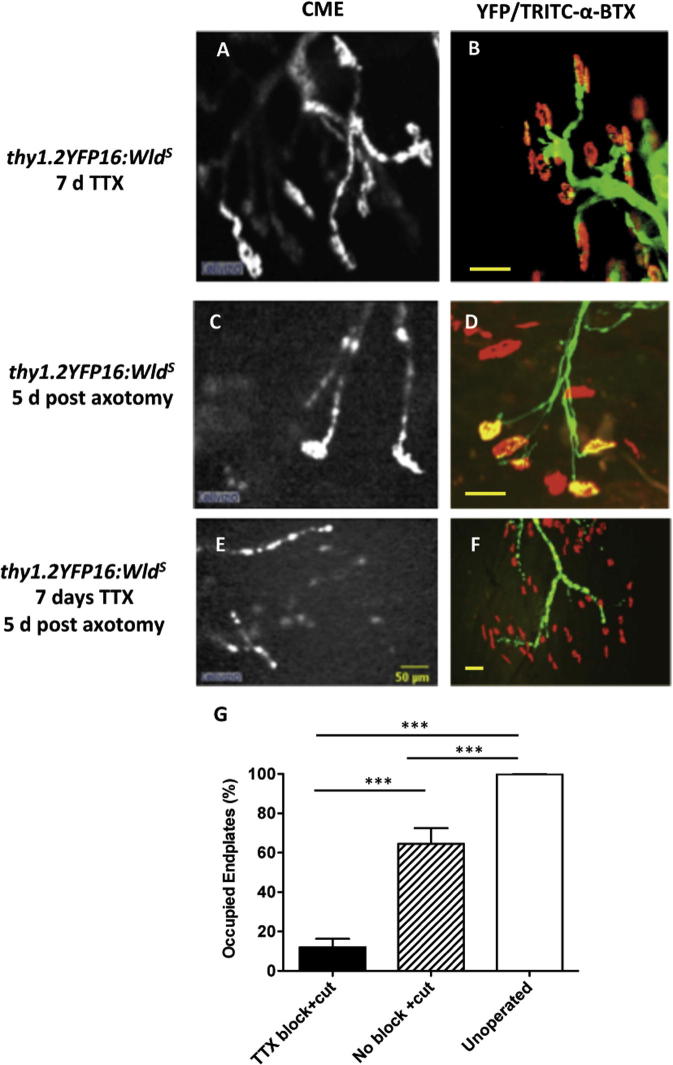
TTX block selectively sensitizes motor nerve terminals. Images obtained *in vivo* using confocal microendoscopy (CME; panels A, C, E) and conventional fluorescence microscopy (B, D, F) from *Wld^S^* mice after TTX block alone (A, B), 5 days after axotomy alone (C, D), or 5 days post axotomy with seven preceding days of complete nerve conduction block with TTX (E, F). Triggering degeneration after 7 days of TTX left most intramuscular axons denuded of their nerve terminals. Images digitally adjusted for overall brightness and contrast only. (G) The number of occupied endplates was significantly reduced in lumbrical muscles 5 days post axotomy from mice preconditioned with 7 days TTX block, compared to untreated axotomized (No block + cut), and unoperated controls (mean ± S.E.M., *n* = 4–7 muscles; *P* < 0.001, ANOVA/Bonferroni).

First, we obtained live images of NMJ in 7 days TTX-blocked/5 days-axotomized muscles of anesthetized *thy1.2YFP16:Wld^S^ in vivo*, using a hand-held confocal microendoscope (CME; (Wong et al., 2009; Brown et al., 2014). In accordance with the physiological data, terminals in control muscles innervated by unsectioned, TTX-blocked nerves were abundant (Fig. 7A), confirming that nerve block alone did not trigger synaptic degeneration. In muscles with nerve section only and no TTX-block, terminals were still readily discernible (Fig. 7C). However, in muscles supplied by sectioned, TTX-blocked nerves, most intramuscular axons were fragmented and motor nerve terminals had degenerated (Fig. 7E). We quantified synaptic degeneration in isolated DL muscle preparations 5 days post axotomy, from images obtained using conventional fluorescence microscopy (Fig. 7B, D, F). In DL muscles from mice with conditioning nerve block and axotomy, significantly fewer motor endplates (TRITC-α-bungarotoxin positive) were either completely or fractionally occupied compared with those following nerve section without preconditioning block. There were about three times as many vacant (denervated) endplates in TTX-blocked muscles: 75.75 ± 3.65% (*n* = 4 mice) of NMJs, compared with only 23.58 ± 7.58% in axotomized-only *Wld^S^* muscles (*n* = 6; *P* < 0.001, ANOVA, Bonferroni post hoc test). The number of vacant (denervated) endplates in contralateral unoperated muscles was negligible (0.07 ± 0.05%, *n* = 7 muscles, Fig. 7G).

### TTX-block selectively sensitized motor nerve terminals

**Fig. 8:**
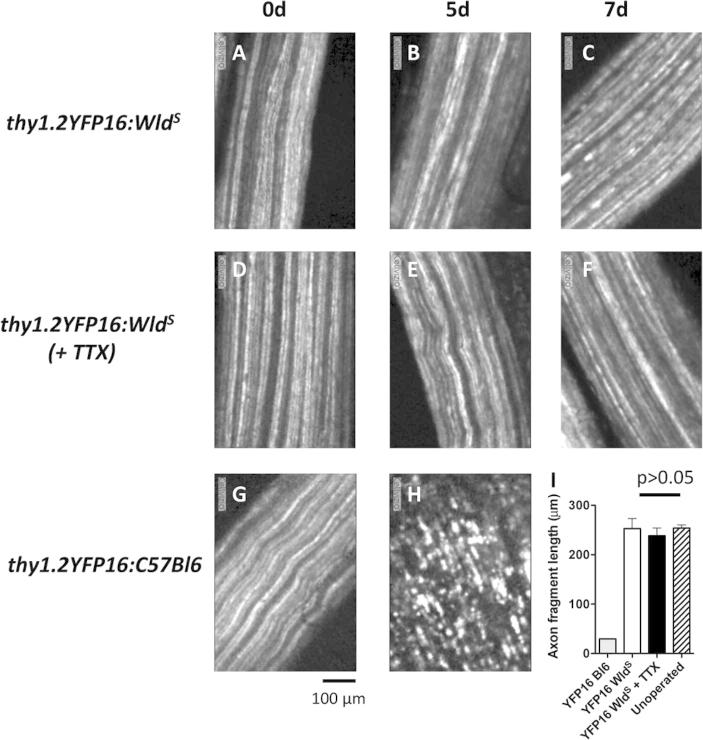
TTX block does not affect axonal integrity. *In vivo*, CME images of tibial nerve axons in *thy1.2YFP16:Wld^S^* mice (A–F) and *thy1.2YFP16:C57Bl6* mice (G, H), up to 7 days post axotomy. (I) there was no difference in axon preservation 5 days post axotomy in TTX-blocked compared with control mice but, as expected, axons in wild-type mice were extensively fragmented. Mean ± S.E.M., *N* = 4 mice in each group, except *thy1.2YFP16:C57Bl6*, wild-type for *Wld^S^* (YFP16Bl6), showing data from a single mouse; (*P* < 0.001; ANOVA/Bonferroni on the other three groups). Images digitally adjusted for overall brightness and contrast only.

Next we asked to what extent motor nerve terminals were selectively affected by axonal conduction block or whether TTX block also sensitized the axons that supplied them. We found that conditioning block did not affect the rate of degeneration of axons. The evidence for this was based on the appearance of axons in tibial nerves of *thy1.2YFP16:Wld^S^* double-mutant mice visualized using CME (Fig. 8A–F). Five days after axotomy there was no discernible difference in the lengths of axon fragments comparing the level of axonal degeneration in saline control-treated *Wld^S^* mice (253 ± 20.46 μm; mean ± SEM; *N* = 4 mice, *n* = 127 axons) and those preconditioned with TTX instillation (238.5 ± 15.49 μm, *N* = 4 mice, *n* = 130 axons respectively, *P* > 0.05; Fig. 8I). Neither was different from the apparent length of axon “segments” imaged in unoperated animals. By contrast, as expected, the distal tibial nerve of one WT (*thy1.2YFP16:C57Bl6*) mouse, axotomized 5 days previously, was copiously populated with short axonal fragments (Fig. 8G–I). Together, these CME imaging data suggest that the sensitizing effects of TTX block on synaptic degeneration occur by a mechanism that is confined to motor nerve terminals.

### TTX-block enhanced neurotransmitter release

Several previous studies have shown that neuromuscular paralysis feeds signals back onto motor nerve terminals, leading to enhanced neurotransmitter release (Snider and Harris, 1979; Tsujimoto et al., 1990; Plomp et al., 1992; Ribchester, 1993; Plomp et al., 1994; Barry and Ribchester, 1995; Davis, 2013). In addition, axotomy sensitizes motor endplates to acetylcholine in *Wld^S^* mouse muscle fibers, enhancing postsynaptic responses to spontaneous transmitter release, most likely due to an activity-dependent change in membrane resistance (Barry and Ribchester, 1995; Ribchester et al., 1995). We therefore asked whether TTX-block administered via the capillary implant method we employed here would also affect neurotransmitter release and other physiological properties of synaptic potentials.

**Fig. 9:**
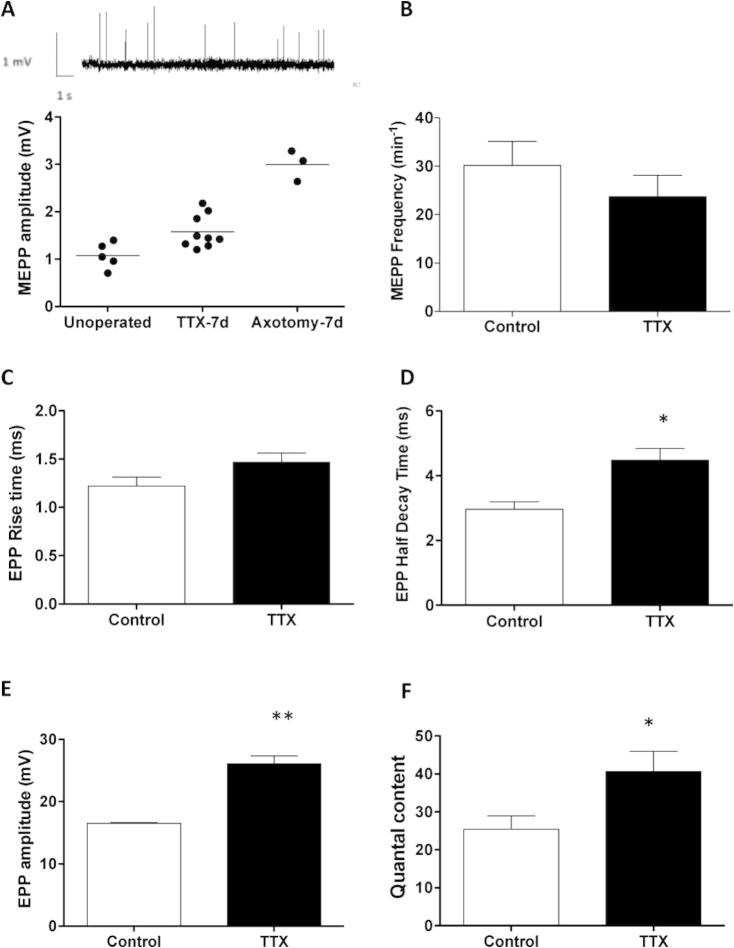
TTX block enhances synaptic transmission. (A) Inset, representative recordings of MEPPs from an FDB muscle fiber of a *Wld^S^* mouse, after 7 days preconditioning, unilateral sciatic nerve block. MEPP amplitude was significantly increased but, interestingly, not as substantially as in remaining innervated fibers 7 days after axotomy, rather than nerve block (*P* < 0.01; ANOVA/Bonferroni comparing all three groups). Neither MEPP frequency (B) nor EPP rise time (C) was significantly affected by nerve block. However, EPP half-decay time (D), EPP amplitude (E), and quantal content (F) were all significantly increased (*P* < 0.05; *t*-tests in each case). Graphs show mean ± S.E.M., *n* = 3–9 muscles in each group. Single asterisks, *P* < 0.05; double asterisks: *P* < 0.01).

We found no significant effect of TTX block on FDB muscle fiber resting membrane potential (TTX-blocked; −67.33 ± 2.38 mV, *n* = 5; saline control: −71.59 ± 2.38 mV, *n* = 3; *P* > 0.05, *t*-test). FDB muscles from *Wld^S^* mice without conditioning nerve block showed a mean MEPP amplitude of 1.08 ± 0.12 mV (*n* = 5; Fig. 9A). Seven days of nerve block caused a significant, approximately 50% increase in MEPP amplitude (1.58 ± 0.12 mV, *n* = 9). However, this increase was less than that observed 7 days after complete axotomy (2.55 ± 0.47 mV, *n* = 3 respectively, *P* < 0.01 ANOVA; Bonferroni post hoc; compared with Ribchester et al., 1995).

By contrast, there was no statistically significant change in MEPP frequency after nerve block (23.68 ± 4.47 MEPPs min^−1^, *n* = 6, compared with controls: 30.18 ± 4.97 MEPPs min^−1^, *n* = 4, *P* > 0.05, *t*-test; Fig. 9B). These data were perhaps surprising given the reported increase in spontaneous MEPP frequency within 1–3 days of chronic paralysis in the mouse extensor digitorum longus (EDL) muscle (Tsujimoto et al., 1990), although the magnitude of the difference in that previous study was quite small and the variability quite large, even after 6 days of TTX block.

TTX block for 7 days also had no significant effect on EPP latency (2.57 ± 0.06 ms *n* = 7 muscles, versus 2.43 ± 0.12 ms, *n* = 5, in controls) or rise time (1.47 ± 0.09 ms, *n* = 5, versus 1.22 ± 0.09 ms, *n* = 3; Fig. 9C). Half-decay time was significantly increased, however (4.47 ± 0.36 ms, *n* = 5, versus 2.96 ± 0.23 ms, *n* = 3: *P* < 0.05, t-test; Fig. 9D). There was also a significant increase in peak amplitude of EPPs (26.10 ± 1.25 mV, *n* = 5, vs. 16.51 ± 0.14 mV, *n* = 3: *P* < 0.01, Fig. 9E) and a significant increase in QC, calculated using the variance method (40.67 ± 5.32 quanta, *n* = 6), compared with controls (25.42 ± 3.26, *n* = 7, *P* < 0.05, *t*-test, Fig. 9F).

These observations support previous studies that demonstrated an effect of chronic nerve conduction block on spontaneous and evoked neurotransmitter release at NMJ under other conditions (Snider and Harris, 1979; Tsujimoto et al., 1990; Barry and Ribchester, 1995). In the context of the present study the data further indicate the sensitizing effect of paralysis on properties of motor nerve terminals.

### TTX-block primed synaptic degeneration in WT mice

Finally, we asked whether paralysis would also affect Wallerian-like degeneration in motor nerve terminals in WT mice; or whether it was a unique effect in mice expressing the chimeric mutant *Wld^S^* gene. Synaptic degeneration is one of the earliest signs of WD and is normally complete by 18 h post axotomy in mice (Slater, 1966; Winlow and Usherwood, 1975; Gillingwater et al., 2002; Wong et al., 2009), whereas the earliest signs of axon fragmentation in the distal stump of an axotomized peripheral nerve are normally discernible only after about 36 h (Beirowski et al., 2004; Beirowski et al., 2005; Wong et al., 2009). In light of this aggressive synaptic pathology, there was only a short time window for obtaining definitive data. Thus, we assayed the amount of synaptic degeneration in FDB muscles of WT (C57Bl6) mice, exactly12 h after cutting the tibial nerve.

**Fig. 10:**
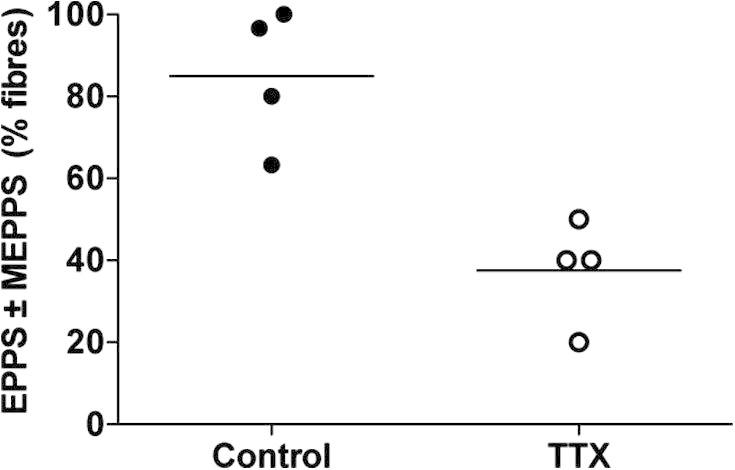
Effect of TTX block on loss of innervation, 12 h after sectioning the sciatic nerve in wild-type mice. Those receiving a preconditioned 7 days of sciatic nerve block showed significantly fewer innervated or responsive NMJs than unblocked controls (*P* < 0.01; *t*-test).

Seven days of preconditioning TTX-block in WT mice significantly increased the amount of synaptic degeneration observed 12 h after nerve section. In control (no block) FDB muscles, 61.67 ± 17.56% of fibers were responsive, compared to 6.25 ± 3.75% in those primed with a conditioning TTX block (*P* = 0.022, *t*-test; *n* = 4 in each group, Fig. 10). Thus, a mechanism by which disuse sensitizes motor nerve terminals to axotomy in *Wld^S^* mice seems likely also to be affected in non-mutant mice.

### Synaptic degeneration was enhanced in mice given prolonged access to running wheels

**Fig. 11:**
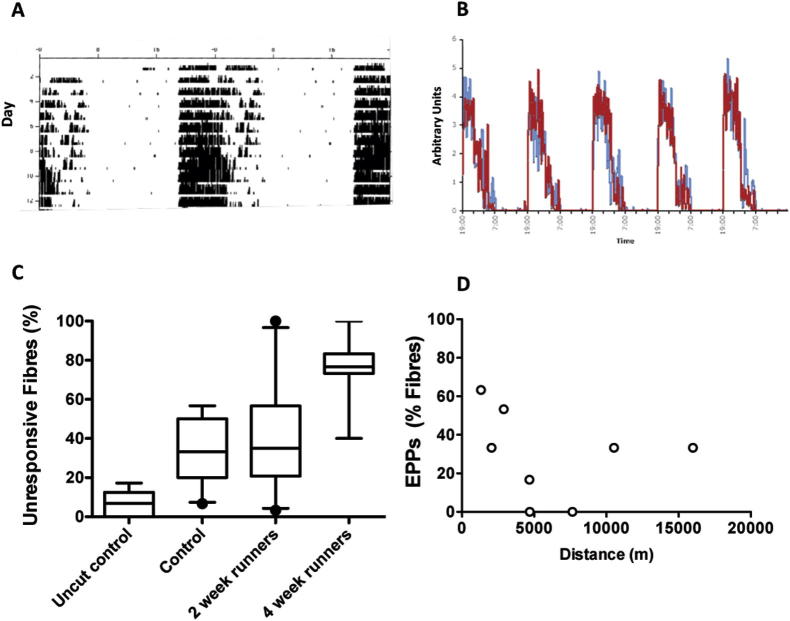
Prolonged self-motivated exercise sensitizes NMJ to axotomy-induced degeneration. (A) Representative circadian activity pattern in a *Wld^S^* mouse. Each horizontal trace is a record of activity over a 24 h period, divided into 12 h of light and 12 h of darkness. Each vertical line is proportional to the number of wheel revolutions. (B) Integrated activity of *Wld^S^* mice (red) and C57Bl6 mice (blue) (*n* = 6 mice in each case) shows no discernible difference in the pattern or amount of circadian activity in the two strains of mice. As expected, most activity was in the dark. (C) Box-whisker plots of incidence (median and interquartile range; outliers >90 percentile shown as dots) of unresponsive FDB muscle fibers (neither MEPPs, nor evoked EPPs observable on tibial nerve stimulation in isolated preparations) in four groups of mice: controls with no nerve section; *Wld^S^* controls with no running wheels, 5 day post-axotomy; *Wld^S^* mice with provision of running wheels for 2 weeks, then 5 days post axotomy; *Wld^S^* mice with provision of running wheels for 4 weeks, then 5 days post axotomy. *n* = 7–11 muscles per group. (D) Plot of incidence of Responsive fibers (evoked EPPs) versus recorded mean diurnal running wheel activity, averaged over 4 weeks and assayed 5 days post axotomy. While there was no clear evidence of regression or correlation, mice running the least distance had stronger levels of synaptic protection than those running the most. (For interpretation of the references to color in this figure legend, the reader is referred to the web version of this article.)

In the third and final group of experiments, we asked whether facilitating endogenous activity might influence subsequent axotomy-induced synaptic degeneration. To test this, we housed *Wld^S^* mice individually in cages fitted with running wheels for either 2 weeks or 4 weeks, before cutting the sciatic nerve unilaterally. Innervation of the FDB muscles was assessed 5 days later. *Wld^S^* and WT C57Bl6 mice showed similar circadian patterns of activity (Fig. 11A, B). During the conditioning period, mice ran 6225 ± 1759 m/day, based on counts of wheel revolutions, but the range of activity varied more than 10-fold: from less than 1.5 km/day to more than 15 km/day. Most of this activity occurred during the controlled 12-h period of darkness.

First, we tested for the possibility that voluntary activity might enhance, rather than reduce synaptic protection. Protection of axons and synapses by *Wld^S^* is strongly “gene-dose” dependent and heterozygotes show strong axon protection but weak or absent synaptic protection (Mack et al., 2001; Wong et al., 2009). Thus, *Wld^S^* heterozygotes constitute a sensitized genetic background for detecting additive protective effects on synaptic degeneration. However, we found that after one-month of conditioning access to running wheels, synaptic transmission was lost from 100% of muscle fibers within 3 days of axotomy in muscles of *Wld^S^*/+ mice, the same as in mice without wheel access (*N* = 18 mice, *n* > 7 muscles in each group; data not shown).

Next we asked whether voluntary activity might enhance degeneration. For these experiments we utilized *Wld^S^* homozygotes (Fig. 1C). As expected, there were very few unresponsive fibers in the uncut control group (6.78 ± 2.32%, *n* = 8, *P* < 0.05, ANOVA/Bonferroni; Fig. 11C). After 2 weeks of activity, the mean number of unresponsive fibers, 5 days after axotomy was not significantly different from that in age-matched homozygous *Wld^S^* mice housed in cages without wheels (40.33 ± 8.81%, *n* = 10 muscles vs. 35.15 ± 5.46%, *n* = 11; *P* > 0.05, ANOVA). However, in mice with 4 weeks of preconditioning activity, the number of unresponsive fibers 5 days post axotomy was almost twice as great as in the control group (75.14 ± 6.79%, *n* = 7, *P* < 0.01, ANOVA/Bonferroni). There was no overt correlation or regression between distance run and the number of responsive fibers after axotomy (Pearson *r* −0.2313, *P* > 0.05; Fig. 11D). However, it is perhaps noteworthy that the mice that ran the least average distance per night showed the highest levels of synaptic protection after axotomy.

In sum, the results of all three groups of experiments showed that either: (a) intensive stimulation *ex vivo*; (b) complete nerve conduction block *in vivo*; or (c) voluntary wheel running over an extended period, all reduced synaptic protection mediated by *Wld^S^* gene expression. Normal levels of endogenous activity may therefore be an obligatory moderating influence on synaptic maintenance and degeneration in this paradigm.

## Discussion

We have presented here new and direct evidence that the rate of neuromuscular synaptic degeneration in response to axotomy is sensitive to activity. The level of axonal or synaptic activity, either before or after axotomy, exerted clear influences on the persistence of axotomized synapses both in WT mice (see Fig. 10) and in the slow degeneration (*Wld^S^*) strain in which axons and synapses are normally protected by expression of a chimeric protein containing the NAD synthetic enzyme Nmnat-1 (Coleman and Freeman, 2010; Gilley and Coleman, 2010; Conforti et al., 2014). Thus, we may infer from the present study that moderate levels of activity serve an important function in neuromuscular synaptic maintenance; but extremes – either complete disuse or sustained high-frequency activation – render motor nerve terminals more vulnerable to potent triggers of degeneration, like axotomy. In light of emerging similarities at a molecular level between synaptic degeneration induced by axotomy or in disease (Gillingwater and Ribchester, 2003; Conforti et al., 2014), our findings are potentially of interest in a disease context as well, since the data are consistent with the notion that activity contributes to synaptic maintenance in general and, conversely, synaptic vulnerability to potent neurodegenerative triggers or risk factors.

Our first group of experiments was motivated in part by our previous study, in which we observed that axotomized sensory terminals innervating muscle spindles, which continued to respond to natural stimulation, were more robustly protected from degeneration in *Wld^S^* mice than motor nerve terminals (Oyebode et al., 2012). We hypothesized that differences in ongoing, endogenous activity in the axotomized sensory and motor axons contributed to the difference in protection. However, the results of stimulation of motor axons in isolated preparations did not wholly support this hypothesis. There was a liminal protective effect of low-frequency stimulation in these experiments but more striking was the fourfold enhancement of degeneration when axons and synapses were activated using patterned high-frequency (100 Hz) stimulation. Reducing the Ca^2+^/Mg^2+^ ratio counteracted the effects of stimulation, consistent with a mechanism that depends on activity-dependent increases in cytoplasmic Ca^2+^. We discuss this further in consideration of the possible mechanisms of synaptic vulnerability to neurodegenerative triggers below.

Our second and third groups of experiments were designed to explore in more detail the relationship between endogenous activity and synaptic protection. The data show that prior levels of axonal or synaptic activity also influence the subsequent rate of synaptic degeneration initiated by a potent trigger: axotomy. The most compelling data were those showing that a sustained, conditioning period of nerve conduction block, either in *Wld^S^* mice or WT mice led to accelerated synaptic degeneration after nerve section, reducing residual innervation to about one-fifth the level in saline vehicle controls. These data are consistent with the popular aphorism “use it, or lose it”, often quoted in the context of activity-dependent mitigation of cognitive or motor decline associated with either natural aging or triggered by risk factors for neurodegenerative disease (Swaab et al., 2002; Kirkinezos et al., 2003; Veldink et al., 2003; Frick and Benoit, 2010; Gordon et al., 2010; Power et al., 2010). Conversely, and unexpectedly, we found that prolonged (4 weeks) volitional activity (wheel running) also sensitized synapses to degeneration induced by axotomy, consistent with some reports, but not others, that hyperactivity sensitizes vulnerable motor neurons and their connections to degeneration (Carrasco et al., 2004; Liebetanz et al., 2004; Mahoney et al., 2004; Lepore et al., 2010; Carrasco et al., 2012; Tran et al., 2014). Interestingly, chronic nerve conduction block appears not to accelerate progression of disease signs or loss of muscle innervation in the SOD1 mouse model of ALS (Carrasco et al., 2012). The disadvantage of this model is the highly variable time course both for onset of signs, progression and survival (Benatar, 2007; Scott et al., 2008), whereas the response of axons or NMJs to axotomy in either WT or *Wld^S^* mice is synchronously initiated in motor units by nerve injury and progresses over a time course that is highly consistent between animals with low variance from onset to conclusion. Nevertheless, further studies are required to establish whether the selective autotomy of motor nerve terminals that evidently occurs at early stages in the progression of some familiar or sporadic forms of ALS is a consequence of focal disease or a trigger for Wallerian-like degeneration of motor nerve terminals, or whether there are molecular mechanisms in common (Frey et al., 2000; Fischer et al., 2004; Schaefer et al., 2005; Conforti et al., 2014).

Previous studies have demonstrated that the axonal- and synaptic-protection phenotype of *Wld^S^* mice is modifiable in several ways. In addition to the sensory/motor differences that prompted the present study (Oyebode et al., 2012), other modifiers of the *Wld^S^* phenotype include the *Wld^S^* gene copy-number (“gene-dose”), age, localization of the protective protein to organelles in the axoplasm or synaptic terminals, and interaction with other proteins of known and unknown identity (Mack et al., 2001; Gillingwater et al., 2002; Beirowski et al., 2009; Conforti et al., 2009; Wong et al., 2009; Babetto et al., 2010; Osterloh et al., 2012; Massoll et al., 2013).

The mechanism of activity-dependent sensitization of synaptic degeneration remains a matter of conjecture. There are several differences in the proteomic fingerprints of synaptic terminals in *Wld^S^* mice compared with WT mice, any of which could be of crucial importance in the mechanism of protection or its modulation, either upstream or downstream of the Nmnat isoforms that insure axonal or synaptic integrity (Wishart et al., 2007). However, several lines of evidence suggest that association of *Wld^S^* protein with specific intracellular organelles is crucial for its protective functions. Mitochondria and endoplasmic reticulum have been implicated, on the basis of experiments that alter targeting of the protein and mimicry of its effects (Beirowski et al., 2009; Conforti et al., 2009; Babetto et al., 2010; Beirowski et al., 2010; Barrientos et al., 2011; Avery et al., 2012; Gillingwater and Wishart, 2013; Milde et al., 2013; Villegas et al., 2014). Recent data suggest a strong connection between the enzymic activity of Wld^S^ protein and sterile alpha and TIR motif-containing protein-1 (Sarm1), and their association with mitochondria may prove to crucial to the mechanism of synaptic maintenance (Osterloh et al., 2012; Massoll et al., 2013; Summers et al., 2014). It has also been shown recently that accumulation of nicotinamide mononucleotide (NMN), the substrate for Nmnat isoforms (and Wld^S^ enzymic activity), may be a toxic agent that precipitates axonal fragmentation at the onset of WD, following a steep decline in axonal Nmnat-2 levels (Gilley and Coleman, 2010; Gilley et al., 2013; Milde et al., 2013; Conforti et al., 2014; Di Stefano et al., 2014). It is perhaps of interest to note in this context that aging mitochondria appear to accumulate in motor nerve terminals, because retrograde transport of mitochondria out of motor terminals is slower than their anterograde transport (Misgeld et al., 2007); and that regeneration of motor nerve terminals, or forced localization of Wld^S^ protein to the cytoplasm in aged *Wld^S^* mice, evidently counteracts the age-dependent weakening of synaptic protection in these mice (Gillingwater et al., 2002; Beirowski et al., 2009). It may be of interest and importance to establish whether activity influences the levels or activity of Nmnat, Nampt, or Sarm-1 in axons and synapses, both in WT and *Wld^S^* mice, as potential explanations for the sensitizing effects of activity shown in the present study.

The disparity between the rate of degeneration of synaptic terminals that we observed in *ex-vivo* nerve-muscle preparations from *Wld^S^* mice compared with the rate routinely observed *in vivo* also begs a number of questions about mechanism (Tsao et al., 1994; Ribchester et al., 1995; Tsao et al., 1999; Gillingwater et al., 2002; Parson et al., 2004; Wishart et al., 2007; Bridge et al., 2009). All the same cell types that could contribute to synaptic degeneration are present in the *ex-vivo* dissections (Court et al., 2008). So why do the NMJs not persist or continue to function in oxygenated bathing medium, even at a lower temperature (32 °C) than normal mammalian core temperature, for as long as they do when naturally perfused by oxygenated blood *in vivo*? Perhaps the trauma of isolation itself constitutes a metabolic challenge to motor nerve terminals that Wld^S^ protein expression in native *Wld^S^* mutant mice is insufficient to resist. The energetics of maintaining a functional synapse evidently place considerable demands on the metabolism of motor nerve terminals (Zhu et al., 2012; Schwarz, 2013). Synaptic mitochondria are particularly sensitive to transient hypoxia, ischemia and Ca^2+^ overload (Brown et al., 2006; David et al., 2007; Misgeld et al., 2007; Nguyen et al., 2009; Nguyen et al., 2011; Nguyen et al., 2012; Talbot et al., 2012). This transient ischemic/hypoxic response is also sensitive to Ca^2+^ (Barrett et al., 2014). It would be interesting to determine to what extent the effects of activity or isolation and *ex-vivo* culture converge on a common mechanism, perhaps involving mitochondria, ROS and succinate levels (Chouchani et al., 2013; Chouchani et al., 2014). Intraterminal Ca^2+^ measurements and their sensitivity to mitochondrial inhibitors, ROS and succinate could be fruitful avenues to explore in seeking resolution of these phenotypic characteristics and their modifiers.

Finally, it is of interest to compare the effects of activity on synaptic degeneration induced by axotomy, shown here, with neuromuscular synapse elimination: the natural process of synaptic remodeling that occurs during normal postnatal development in rodents, or in adults after nerve regeneration (Gillingwater and Ribchester, 2001, 2003). Paralysis retards, and high-frequency stimulation accelerates synapse elimination; and this effect is partly dependent on Ca-activation of proteases (O’Brien et al., 1978; Thompson et al., 1979; Taxt, 1983; Thompson, 1983; Connold et al., 1986). Complete paralysis thus prolongs the period when muscle fibers are polyneuronally innervated, sometimes even after activity has resumed (Brown et al., 1982; Barry and Ribchester, 1995; Costanzo et al., 1999, 2000). Paralysis also promotes reactive growth, rather than degeneration, of nerve terminal sprouts (Brown and Ironton, 1977; Betz et al., 1980b; Tsujimoto et al., 1990; Costanzo et al., 2000). Both characteristics, synapse elimination and terminal sprouting, therefore are modulated by activity in a fashion that appears to be opposite to its effects on axotomy-induced degeneration that we have described in the present study. However, this interpretation is complicated by findings that differences in activity brought about by selective nerve conduction block, or by differential stimulation, promotes withdrawal of the disused or less active axons (Ribchester and Taxt, 1983, 1984; Ridge and Betz, 1984; Favero et al., 2010; Favero et al., 2012). Thus, while the triggers for synaptic degeneration after injury, in disease, or during development may differ, the present findings do not rule out that activity-dependent mechanisms that remove synaptic terminals from NMJs, by degeneration or controlled withdrawal, may ultimately converge (Raff et al., 2002; Gillingwater and Ribchester, 2003; Conforti et al., 2014).

## Conclusion

Hitherto, use or disuse was thought to influence synaptic maintenance and mitigate synaptic degeneration but the evidence for this had been indirect. Here we have newly shown, by applying direct methods, that patterned stimulation, chronic disuse, or natural usage of NMJs in adult *Wld^S^* mice strongly influences their vulnerability to axotomy: a potent trigger of synaptic degeneration. We found that moderate levels of activity either before or after disrupting the functional integrity of axons optimized synaptic maintenance and resistance to degeneration, while complete disuse or intensive stimulation increases the rate of synaptic degeneration. The *ex-vivo* preparation that we utilized in the first part of this report enables control over both the activity of NMJs and the composition of the cellular environment. This could prove crucial for further analysis of the mechanisms of activity dependence, or other influences, on synaptic degeneration.

## Author contributions

RRR, RB, AH-A: Designed the studies; performed experiments and analyzed the data shown in Figs. 1, 4 and 6–11; co-wrote the manuscript.

AS: Conducted experiments and analyzed the data shown in Fig. 5.

KD: Carried out experiments and analyzed some of the data included in Fig. 4.

THG: Performed surgery and obtained and analyzed data included in Figs. 2 and 3.
